# Requirement of ATR for maintenance of intestinal stem cells in aging *Drosophila*

**DOI:** 10.18632/aging.100743

**Published:** 2015-05-12

**Authors:** Joung-Sun Park, Hyun-Jin Na, Jung-Hoon Pyo, Ho-Jun Jeon, Young-Shin Kim, Mi-Ae Yoo

**Affiliations:** ^1^ Department of Molecular Biology, Pusan National University, Busan, 609-735, Republic of Korea

**Keywords:** Drosophila, intestinal stem cell, aging, ATM/ATR, DNA damage response

## Abstract

The stem cell genomic stability forms the basis for robust tissue homeostasis, particularly in high-turnover tissues. For the genomic stability, DNA damage response (DDR) is essential. This study was focused on the role of two major DDR-related factors, ataxia telangiectasia-mutated (ATM) and ATM- and RAD3-related (ATR) kinases, in the maintenance of intestinal stem cells (ISCs) in the adult *Drosophila* midgut. We explored the role of ATM and ATR, utilizing immunostaining with an anti-pS/TQ antibody as an indicator of ATM/ATR activation, γ-irradiation as a DNA damage inducer, and the UAS/GAL4 system for cell type-specific knockdown of ATM, ATR, or both during adulthood. The results showed that the pS/TQ signals got stronger with age and after oxidative stress. The pS/TQ signals were found to be more dependent on ATR rather than on ATM in ISCs/enteroblasts (EBs). Furthermore, an ISC/EB-specific knockdown of ATR, ATM, or both decreased the number of ISCs and oxidative stress-induced ISC proliferation. The phenotypic changes that were caused by the ATR knockdown were more pronounced than those caused by the ATM knockdown; however, our data indicate that ATR and ATM are both needed for ISC maintenance and proliferation; ATR seems to play a bigger role than does ATM.

## INTRODUCTION

Adult stem cells perform critical functions in tissue maintenance, particularly in high-turnover tissues such as the intestine [1]. The genome of adult stem cells of organisms with long lifespans is more vulnerable to endogenous and exogenous genotoxic stressors such as reactive oxygen species and radiation [2].

Although the DNA damage response (DDR) is known as a key process in the upkeep of genomic stability [3], the role of major DDR-related factors in the maintenance of adult stem cells has not been fully explored. Kim et al. [3] showed that ataxia telangiectasia-mutated (ATM) and ATM- and RAD3-related (ATR) kinases are key players in DDR. Kim et al. [3] found that both ATM and ATR phosphorylate their substrates, including H2AX and p53, preferentially on a serine or threonine preceding a glutamine (pS/TQ).

This substrate specificity is evolutionally conserved in a wide variety of organisms ranging from yeast to mammals, as demonstrated in experiments with an anti-pS/TQ antibody [4-6].

ATM and ATR share many biochemical and functional similarities in the DDR pathway, including activation by the Mre11-Rad50-Nbs1 complex [7]. Nonetheless, differences between ATM and ATR also exist: ATM is primarily activated by DNA double-strand breaks (DSBs), whereas ATR is responsive to a broad spectrum of DNA damage, particularly that interfering with DNA replication [8]. ATM is involved in the G1/S checkpoint [9], activates the p53 response to DNA damage [10], and interacts with ATR [11]. ATR is essential for the repair of damaged replication forks [8, 12-13]. The ATR-mediated pathway is associated with the G2/M checkpoint [11]. The differences between ATM and ATR may be related to the DDR in tissue-resident stem cells, particularly in high-turnover tissues, for example, the intestinal stem cells (ISCs) of adult *Drosophila* (used in the present study). Despite the importance of intestinal health, which has a significant impact on the lifespan at the organismal level [14], the role of ATM and ATR in ISC homeostasis remains poorly understood.

The *Drosophila* midgut is a widely accepted model for studies of stem cells [15-18]. *Drosophila* ISCs are the only mitotic cells in the adult midgut [15-16]. *Drosophila* ISCs generate two types of differentiated progeny: enterocytes (ECs) and enteroendocrine cells (EEs) through enteroblasts (EBs), which are similar to the cells of mammalian intestines [15-17]. These cell types are distinguished by the expression of specific markers [15-17].

Several key signaling pathways that are involved in the regulation of *Drosophila* ISC proliferation have been identified [18]. Midgut ISCs are sensitive to intrinsic and extrinsic oxidative stress, including aging [19-22]. The aged midgut shows the maintenance of stem cell numbers and increased proliferation of stem cells [19-21, 23]. We recently used γH2AvD (*Drosophila* γH2AX) signals, a marker of DSBs, to monitor accumulation of DNA damage in midgut ISCs in relation to age and oxidative stress [24]. Taken together, these findings suggest that the *Drosophila* midgut is useful for studying the role of DDR-related factors in the ISC maintenance and proliferation. In the present study, we focused on the role of intrinsic ATR and ATM in ISC maintenance and proliferation in the adult *Drosophila* midgut.

## RESULTS

### The pS/TQ signals in *Drosophila* ISCs/EBs increase with age and under the influence of oxidative stress

**Figure 1:**
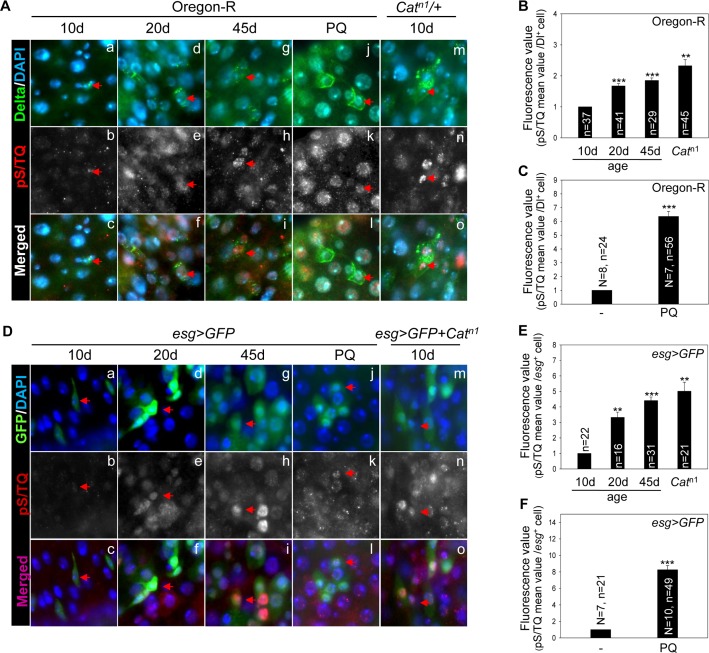
The pS/TQ signal increases with age and under the influence of oxidative stress in *Drosophila* intestinal stem cells (ISCs) (**A**) The age- and oxidative stress-induced increase in pS/TQ signals in Delta protein-positive (Dl^+^) small cells. (a–i) An age-related increase of pS/TQ signals in Dl^+^ small cells. The gut of 10- (a–c), 20- (d–f), and 45-day-old (g–i) wild-type flies was stained with anti-Dl (green) and anti-pS/TQ (red) antibodies and 4′,6-diamidino-2-phenylindole (DAPI; blue). (j–o) An oxidative stress-induced increase in pS/TQ signals in Dl^+^ small cells. The gut of 10-day-old 10 mM PQ-fed (j–l) and *Cat^n1^*/+ (m–o) flies was labeled with anti-Dl (green) and anti-pS/TQ (red) antibodies and DAPI (blue). Red arrows indicate Dl^+^ small cells. c, f, i, l, and o are merged images. The original magnification is 400×. (**B**–**C**) The florescence value of pS/TQ signals in Dl^+^ small cells as a function of age and oxidative stress. The gut of 10-, 20-, and 45-day-old wild-type flies and 10-day-old *Cat^n1^*/+ (B) and 10 mM PQ-fed (**C**) flies was labeled with anti-GFP (green) and anti-pS/TQ (red) antibodies and DAPI (blue). The fluorescence intensity of pS/TQ signals in the Dl^+^ cells was measured in the 5a and b regions of the posterior midgut. The variable *n* indicates the number of Dl^+^ cells. Fluorescence intensity of the cells, which are exactly in focus, was measured in approximately 10 to 14 midgut specimens; ***p < 0.0001, **p < 0.001. (**D**) An age- and oxidative stress-induced increase in pS/TQ signals in *esg*^+^ small cells. (a–i) The age-related increase in pS/TQ signals in *esg*^+^ small cells. The gut of 10- (a–c), 20- (d–f), and 45-day-old (g–i) *esg>GFP* flies was labeled with anti-GFP (green) and anti-pS/TQ (red) antibodies and DAPI (blue). (j–o) The oxidative stress-induced increase in pS/TQ signals in *esg*^+^ small cells. The gut of 10-day-old *esg>GFP* 10 mM PQ-fed (j–l) and *esg>GFP*+*Cat^n1^* (m–o) flies was labeled with anti-GFP (green) and anti-pS/TQ (red) antibodies and DAPI (blue). Red arrows indicate *esg*^+^ cells. c, f, i, l, and o are merged images. The original magnification is 400×. (**E**–**F**) The fluorescence values of pS/TQ signals in *esg*^+^ small cells as a function of age and oxidative stress. The gut of 10-, 20-, and 45-day-old *esg>GFP* and 10-day-old *esg>GFP*+*Cat^n1^* (E) and 10 mM PQ-fed (F) flies was labeled with anti-GFP (green) and anti-pS/TQ (red) antibodies and DAPI (blue). The fluorescence intensity of pS/TQ signals in *esg*^+^ cells was measured in the 5a and b regions of the posterior midgut. The variable *n* indicates the number of *esg*^+^ cells; ***p < 0.0001, **p < 0.001.

We first tested whether the pS/TQ signal (immunostaining), a known marker of ATM/ATR activation in several organisms, is detected in *Drosophila* midgut cells and is modulated with age and by oxidative stress. The pS/TQ signal was detected weakly in Dl^+^ cells (ISCs) in the gut from 10-day-old wild-type flies (Figure 1A a-c, Figure Supplemental S1A). Nonetheless, it increased in an age-dependent manner in Dl^+^ cells in the gut from 20- (Figure 1A d-f) and 45-day-old (Figure 1A g-i) wild-type flies. A strong pS/TQ signal was also detected in Dl^+^ cells of 10-day-old *Cat^n1^/+* and PQ-treated flies (Figure 1A j-o). Quantitative pS/TQ signals in Dl^+^ cells of the gut from 20- and 45-day-old wild type, 10-day-old *Cat^n1^/+*, and PQ-treated flies were increased 1.6-, 1.8-, 2.25-, and 6.2-fold, respectively, compared to 10-day-old wild-type flies (Figure 1B and 1C).

An age-related increase in pS/TQ signals was also detected in *esg*^+^ small cells (ISCs/EBs) of the posterior midgut of 10-, 20-, and 45-day-old *esg>GFP* flies, in 10-day-old *esg>GFP+Cat^n1^*, and PQ-treated flies (Figure 1D). Quantitative pS/TQ signal levels in *esg*^+^ cells of 20- and 45-day-old wild-type, 10-day-old *esg>GFP+Cat^n1^*, and PQ-treated flies were increased 3.2-, 4.2-, 5.1-, and 8.1-fold, respectively, compared to 10-day-old wild-type flies (Figure 1E and 1F). We also analyzed pS/TQ signals in differentiated cells and observed a few increase of the signal in ECs and EEs with age and oxidative stresses (Figure Supplemental S2). These results meant an increased pS/TQ signal in ISCs of the gut affected by age- and oxidative stress.

### DNA damage-induced pS/TQ signals in *Drosophila* ISCs/EBs is more dependent on ATR than on ATM

**Figure 2:**
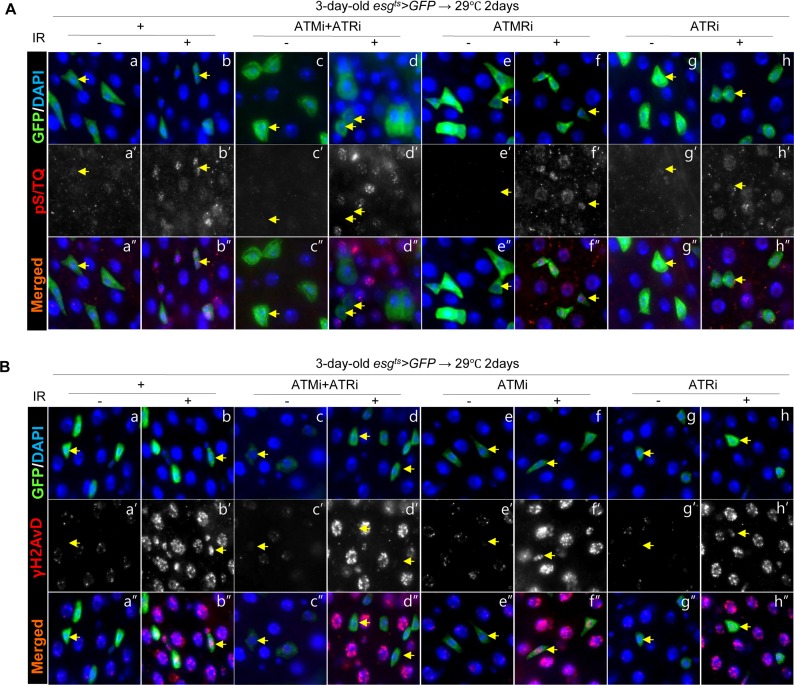
Effects of the intestinal stem cell (ISC)/enteroblast (EB)-specific ATM/ATR knockdown on pS/TQ and γH2AvD signals after γ-irradiation (**A**) The pS/TQ signals after γ-irradiation in the midgut with an ISC/EB-specific knockdown of ATM, ATR, or both. Flies carrying *esg^ts^>GFP* (a–b''), *esg^ts^>GFP*+*ATMi*+*ATRi* (c–d''), *esg^ts^>GFP*+*ATMi* (e–f''), or *esg^ts^>GFP*+*ATRi* genotype (g–h'') were kept at 29 °C for 2 days and then exposed to γ-radiation. One hour after the irradiation, the gut of unirradiated (a, c, e, and g) and irradiated (b, d, f, and h) flies was excised and labeled with anti-GFP (green) and anti-pS/TQ (red) antibodies and 4′,6-diamidino-2-phenylindole (DAPI, blue). a''–h'' are merged images. (**B**) The γH2AvD signals after the irradiation in the midgut with an ISC/EB-specific knockdown of ATM, ATR, or both. Flies carrying the *esg^ts^>GFP* (a–b''), *esg^ts^>GFP*+*ATMi*+*ATRi* (c–d''), *esg^ts^>GFP*+*ATMi* (e–f''), or *esg^ts^>GFP*+*ATRi* genotype (g–h'') were kept at 29 °C for 2 days and exposed to γ-radiation. One hour after the irradiation, the gut of unirradiated (a, c, e, and g) and irradiated (b, d, f, and h) flies was excised and labeled with anti-GFP (green) and anti-γH2AvD (red) antibodies and DAPI (blue). a''–h'' are merged images. The original magnification is 400×.

We determined whether the pS/TQ signal is associated with DNA damage and is a dependable indicator of ATM/ATR activity in the adult *Drosophila* midgut by means of ionizing radiation (IR) as a DNA damage inducer and by means of flies with an ISC/EB-specific knockdown of ATM, ATR, or both under the *esg^ts^>GFP* genotype. We examined pS/TQ signal strength in *esg*^+^ cells of the gut from *esg^ts^>GFP*, *esg^ts^>GFP+ATMi*+*ATRi*, *esg^ts^>GFP+ATRi*, and *esg^ts^>GFP+ATMi* flies 1 h after application of 5 Gy of γ-ray irradiation. Strong pS/TQ signals were detected in ISCs and EBs as well as ECs in irradiated *esg^ts^>GFP* wild-type flies in contrast to the weak pS/TQ signals in the midgut cells of unirradiated flies (Figure 2A a-a'' and b-b''). The increase in the pS/TQ signal that was induced by γ-irradiation indicated that the pS/TQ signal was associated with DNA damage.

In contrast to the signal in wild type *esg^ts^>GFP* flies, the γ-irradiation-induced increase of the pS/TQ signal was greatly reduced in *esg*^+^ cells (ISC/EB) of *esg^ts^>GFP+ATMi+ATRi* flies with an ISC/EB-specific knockdown of both ATM and ATR (Figure 2A c-d''). The IR-induced increase in pS/TQ signals was also greatly decreased specifically in *esg*^+^ cells of the gut of *esg^ts^>GFP+ATRi* flies with an ISC/EB-specific ATR knockdown (Figure 2A g-h'') and mildly decreased in *esg*^+^ cells of the *esg^ts^>GFP+ATMi* gut (Figure 2A e-f'').

We also examined γH2AvD foci in *esg*^+^ cells of the gut from *esg^ts^>GFP*, *esg^ts^>GFP+ATMi+ATRi*, *esg^ts^>GEP+ATRi* and *esg^ts^>GFP+ATMi* flies 1 h after administration of 5 Gy of γ-irradiation. As expected, strong γH2AvD signals were detected in midgut cells including ISCs/EBs in irradiated *esg^ts^>GFP* wild type flies (Figure 2B a-b''); however, the IR-induced increase in the γH2AvD signal was strongly decreased specifically in *esg*^+^ cells of the gut from *esg^ts^>GFP+ATMi+ATRi* flies (Figure 2B c-d''). The IR-induced increase in the γH2AvD signal was also greatly decreased specifically in *esg*^+^ cells of the gut from *esg^ts^>GFP+ATRi* flies (Figure 2B g-h'') and mildly decreased in *esg*^+^ cells of *esg^ts^>GFP+ATMi* gut specimens (Figure 2B e-f'').

These results indicated that the pS/TQ signal that was induced by γ-irradiation in the ISCs/EBs of the *Drosophila* midgut was dependent on ATM/ATR, and the role of ATR was more important than that of ATM.

**Figure 3:**
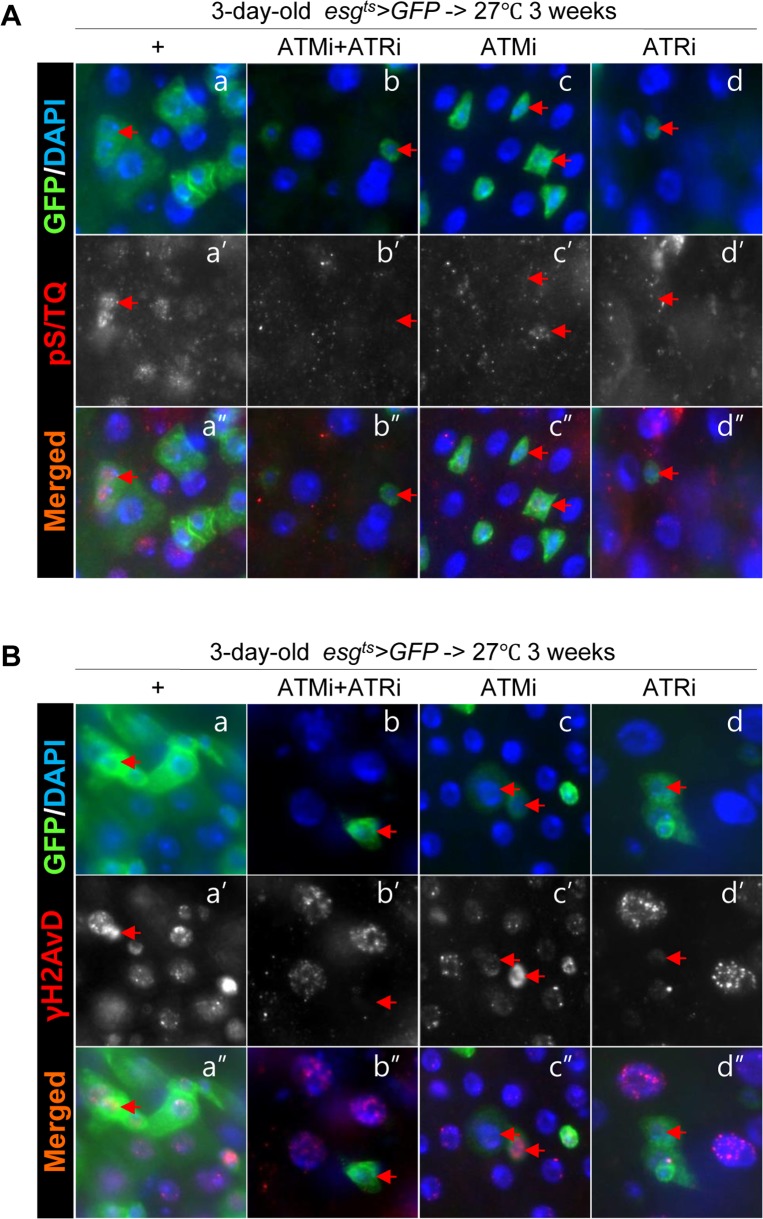
Effects of the intestinal stem cell (ISC)/enteroblast (EB)-specific knockdown of ATM or ATR on activation of the DNA damage response (DDR) with age (**A**) Effects of the ISC/EB-specific knockdown of ATM, ATR, or both on the age-induced increase of pS/TQ signals. The gut specimens of *esg^ts^>GFP*, *esg^ts^>GFP+ATMi+ATRi*, *esg^ts^>GFP+ATMi*, and *esg^ts^>GFP+ATRi* flies were cultured at 27 °C for 3 weeks, and were labeled with anti-pS/TQ (red) and anti-GFP (green) antibodies and 4′,6-diamidino-2-phenylindole (DAPI, blue). (**B**) Effects of the ISC/EB-specific knockdown of ATM, ATR, or both on the age-induced increase of γH2AvD signals. The gut specimens of *esg^ts^>GFP*, *esg^ts^>GFP+ATMi+ATRi*, *esg^ts^>GFP+ATMi*, and *esg^ts^>GFP+ATRi* flies were cultured at 27 °C for 3 weeks, and were labeled with anti-γH2AvD (red) and anti-GFP (green) antibodies and 4′,6-diamidino-2-phenylindole (DAPI, blue). Arrows indicate *esg*^+^ small cells. The original magnification is 400×.

Next, to address whether the age-related increase of pS/TQ signal is dependent on ATM/ATR, we checked changes of the pS/TQ signal with age in *esg*^+^ cells of the gut from *esg^ts^>GFP*, *esg^ts^>GFP+ATMi+ATRi*, *esg^ts^>GFP+ATMi*, and *esg^ts^>GFP+ATRi* flies for 3 weeks at 27 °C since most of stem cells (*esg*^+^) in the posterior midgut were disappeared after 2 weeks at 29 °C. In aged gut specimens, a strong pS/TQ signal was detected in *esg*^+^ small cells of *esg^ts^>GFP* flies (Figure 3A a-a'', Figure Supplemental S3), but the age-related increase of pS/TQ signals was not detected in *esg*^+^ small cells of *esg^ts^>GFP+ATMi+ATRi* flies (Figure 3A b-b''). The age-relaed increase of pS/TQ signals was also greatly decreased specifically in *esg*^+^ cells of the gut specimens from *esg^ts^>GFP+ATRi* flies (Figure 3A d-d'') and mildly decreased in *esg*^+^ cells of *esg^ts^>GFP+ATMi* gut specimens (Figure 3A c-c''). We also checked changes of the γH2AvD signal with age in *esg^+^* cells of gut specimens from *esg^ts^>GFP*, *esg^ts^>GFP+ATMi+ATRi*, *esg^ts^>GFP+ATMi* and *esg^ts^>GFP+ATRi* flies. In gut specimens from aged flies, a strong γH2AvD signal was detected in *esg*^+^ small cells of *esg^ts^>GFP* flies (Figure 3B a-a''). Nonetheless, the age-related increase of γH2AvD signals was not detected in the gut from *esg^ts^>GFP+ATMi+ATRi* flies (Figure 3B b-b''). The age-related increase of γH2AvD signals was also strongly decreased specifically in *esg^+^* cells of the gut from *esg^ts^>GFP+ATRi* flies (Figure 3B d-d'') and mildly decreased in *esg^+^* cells of *esg^ts^>GFP+ATMi* gut specimens (Figure 3B c-c'').

**Figure 4:**
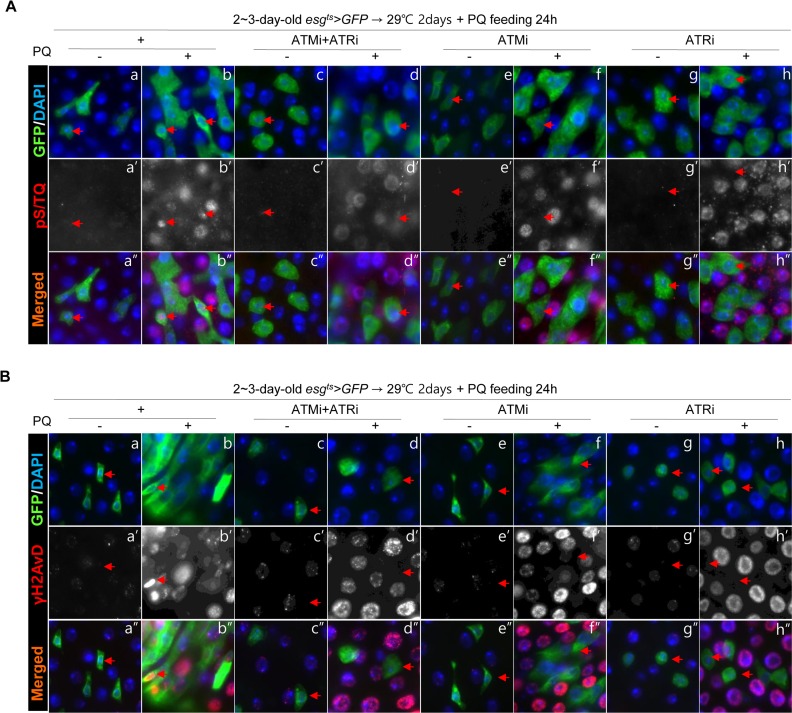
Effects of the intestinal stem cell (ISC)/enteroblast (EB)-specific knockdown of ATM or ATR on activation of the DNA damage response (DDR) by oxidative stress (**A**) Effects of the ISC/EB-specific knockdown of ATM, ATR, or both on the oxidative stress-induced increase of pS/TQ signals. The gut specimens of *esg^ts^>GFP*, *esg^ts^>GFP*+*ATMi*+*ATRi*, *esg^ts^>GFP*+*ATMi*, and *esg^ts^>GFP*+*ATRi* flies (kept at 29 °C for 2 days, with subsequent feeding on media containing 10 mM PQ for 1 day) were labeled with anti-pS/TQ (red) and anti-GFP (green) antibodies and 4′,6-diamidino-2-phenylindole (DAPI, blue). (**B**) Effects of the ISC/EB-specific knockdown of ATM, ATR, or both on the oxidative stress-induced increase of γH2AvD signals. The gut specimens of *esg^ts^>GFP*, *esg^ts^>GFP*+*ATMi*+*ATRi*, *esg^ts^>GFP*+*ATMi*, and *esg^ts^>GFP*+*ATRi* flies (kept at 29 °C for 2 days, with subsequent feeding on media containing 10 mM PQ for 1 day) were labeled with anti-γH2AvD (red) and anti-GFP (green) antibodies and DAPI (blue). Arrows indicate *esg*^+^ small cells. The original magnification is 400×.

In addition, we checked changes of the pS/TQ signal after PQ treatment in *esg*^+^ cells of the gut from *esg^ts^>GFP*, *esg^ts^>GFP+ATMi+ATRi*, *esg^ts^>GFP+ATMi,* and *esg^ts^>GFP+ATRi* flies. In PQ-treated gut specimens, a strong pS/TQ signal was detected in *esg*^+^ small cells of *esg^ts^>GFP* flies (Figure 4A a-b''), but the PQ-induced increase of pS/TQ signals was not detected in *esg*^+^ small cells of *esg^ts^>GFP+ATMi+ATRi* flies (Figure 4A c-d''). The PQ-induced increase of pS/TQ signals was also greatly decreased specifically in *esg*^+^ cells of the gut specimens from *esg^ts^>GFP+ATRi* flies (Figure 4A g-h'') and mildly decreased in *esg*^+^ cells of *esg^ts^>GFP+ATMi* gut specimens (Figure 4A e-f''). We also checked changes of the γH2AvD signal after PQ treatment in *esg*^+^ cells of gut specimens from *esg^ts^>GFP*, *esg^ts^>GFP+ATMi+ATRi*, *esg^ts^>GFP+ATMi* and *esg^ts^>GFP+ATRi* flies. In gut specimens from PQ-treated flies, a strong γH2AvD signal was detected in *esg*^+^ small cells of *esg^ts^>GFP* flies (Figure 4B a-b''). Nonetheless, the PQ-induced increase of γH2AvD signals was not detected in the gut from *esg^ts^>GFP+ATMi+ATRi* flies (Figure 4B c-d''). The PQ-induced increase of γH2AvD signals was also strongly decreased specifically in *esg*^+^ cells of the gut from *esg^ts^>GFP+ATRi* flies (Figure 4B g-h'') and mildly decreased in *esg*^+^ cells of *esg^ts^>GFP+ATMi* gut specimens (Figure 4B e-f'').

These results indicated that the pS/TQ signal that was induced with age and by oxidative stress in the ISCs/EBs of the *Drosophila* midgut was dependent on ATM/ATR, and the role of ATR was more important than that of ATM.

Taken together, these results indicated that the pS/TQ signal that was induced by DNA damage in the ISCs/EBs of the *Drosophila* midgut was dependent on ATM/ATR, and the role of ATR was more crucial than that of ATM.

### A knockdown of ATM/ATR in ISCs/EBs affects maintenance and proliferation of midgut ISCs

To understand the role of the age-related increase in pS/TQ signals (ATM/ATR activity) in midgut ISCs, we characterized the effects of a cell type-specific persistent knockdown of ATM, ATR or both in the posterior midgut. This knockdown was expressed in ISCs/EBs using *esg^ts^>GFP* flies kept at 29 °C for 7 days.

**Figure 5:**
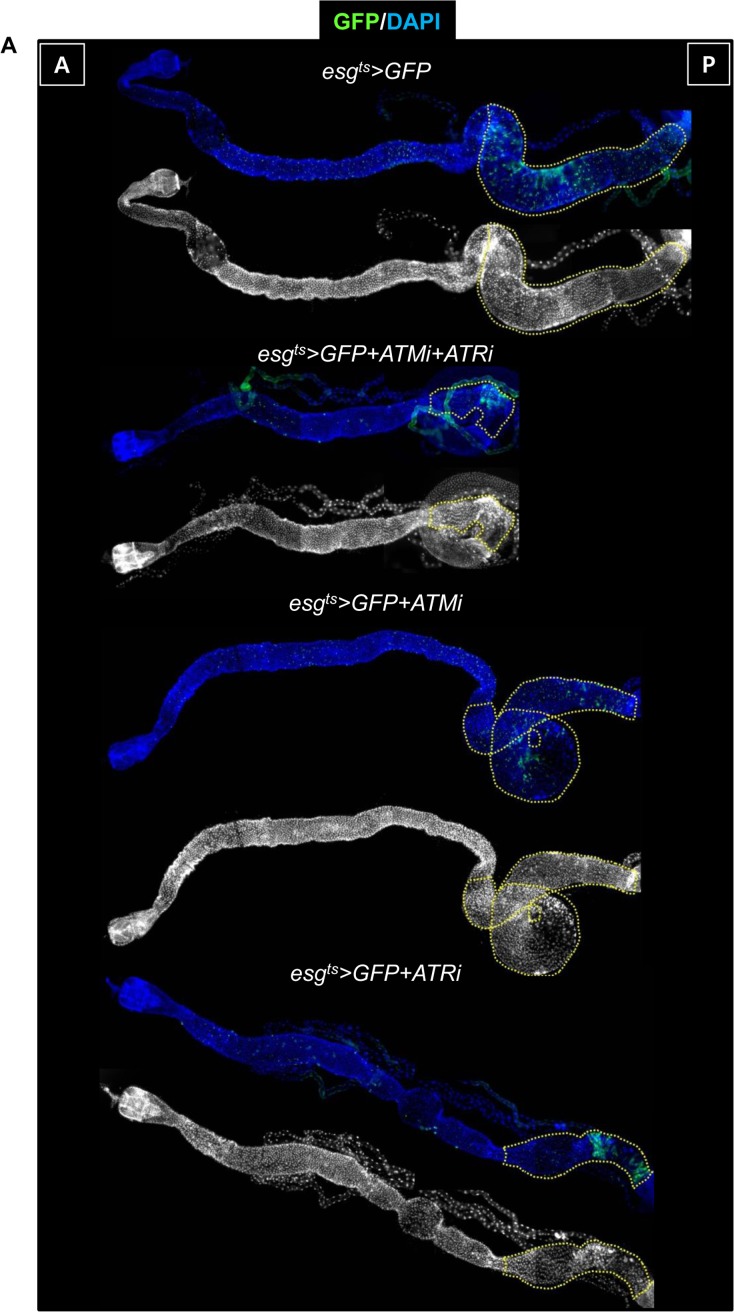

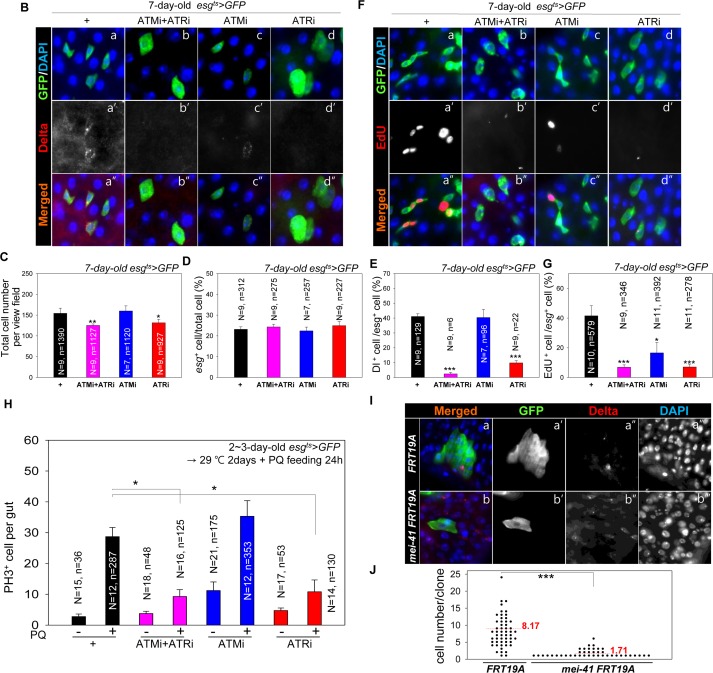
ATM and ATR are required for proliferation and maintenance of *Drosophila* intestinal stem cells (ISCs) (**A**) The phenotype of the midgut with an ISC/enteroblast (EB)-specific knockdown of ATM, ATR, or both. The gut specimens of *esg^ts^>GFP*, *esg^ts^>GFP*+*ATMi+ATRi*, *esg^ts^>GFP*+*ATMi*, or *esg^ts^>GFP*+*ATRi* flies (kept at 29 °C for 14 days) were labeled with an anti-GFP (green) antibody and 4′,6-diamidino-2-phenylindole (DAPI, blue). The yellow dots indicate the posterior region. The regions with yellow dots show a large *esg-GFP*^+^ population in a particular region. The original magnification is 100×. (**B**) A loss of Delta protein-positive (Dl^+^) cells in the midgut with an ISC/EB-specific knockdown of ATM, ATR, or both. The gut specimens of *esg^ts^>GFP* (a-a''), *esg^ts^>GFP*+*ATMi+ATRi* (b-b''), *esg^ts^>GFP*+*ATMi* (c-c''), or *esg^ts^>GFP*+*ATRi* flies (d-d'') (kept at 29 °C for 7 days) were labeled with anti-GFP (green) and anti-Dl (red) antibodies and DAPI (blue). a''–d'' are merged images. The original magnification is 400×. (**C**) A graph showing the total cell number in the gut with an ISC/EB-specific knockdown of ATM, ATR, or both. N: the number of gut specimens, n: the total cell number. *p < 0.01. **p < 0.001. (**D**) A graph showing the the ratio of *esg*^+^ cells to total cells in the midgut with an ISC/EB-specific knockdown of ATM, ATR, or both. N: the number of gut specimens, N: the number of gut specimens, n: the *esg*^+^ cell number. (**E**) A graph showing the ratio of Dl^+^ cells to *esg*^+^ cells in the midgut with an ISC/EB-specific knockdown of ATM and ATR. N: the number of gut specimens, n: the Dl^+^ cell number. ***p < 0.0001. The numbers of cells of each cell type were counted in the R5 region of the posterior midgut under a microscope. (**F**) The gut specimens of *esg^ts^>GFP* (a), *esg^ts^>GFP*+*ATMi+ATRi* (b), *esg^ts^>GFP*+*ATMi* (c), or *esg^ts^>GFP*+*ATRi* flies (d) (kept at 29 °C for 7 days with subsequent feeding on EdU-containing media for 24 h) were stained with anti-GFP (green) and anti-EdU (red) antibodies and DAPI (blue). The original magnification is 400×. (**G**) A graph showing the ratio of EdU^+^ cells to *esg*^+^ cells in the midgut with an ISC/EB-specific knockdown of ATM, ATR, or both. The numbers of cells of each cell type were counted in the R5 region of the midgut. N: the number of gut specimens, n: the *esg*^+^ cell number. *p < 0.01. ***p < 0.0001. (**H**) Effects of the ISC/EB-specific knockdown of ATM, ATR, or both on the oxidative stress-induced increase in proliferation of ISCs. The gut specimens of *esg^ts^>GFP* (black), *esg^ts^>GFP*+*ATMi*+*ATRi* (pink), *esg^ts^>GFP*+*ATMi* (blue), and *esg^ts^>GFP*+*ATRi* (red) flies (kept at sub 29 °C for 2 days, with subsequent feeding on media containing 10 mM PQ for 24 h) were labeled with anti-PH3 (red) and anti-GFP (green) antibodies and DAPI (blue). The numbers of PH3^+^ cells were counted in the whole midgut. The data (mean ± SE) from *esg^ts^>GFP* (PQ-/+), *esg^ts^>GFP*+*ATMi*+*ATRi* (PQ-/+), *esg^ts^>GFP*+*ATMi* (PQ-/+), and *esg^ts^>GFP*+*ATRi* (PQ-/+) flies were collated from 15, 12, 18, 16, 21, 12, 17, and 14 gut specimens, respectively. N: the number of gut specimens, n: the mitotic cell number; *p < 0.01. (I) Effect of *mei-41* null mutant MARCM on ISC maintenance of posterior midguts. The flies carrying *hsFLP, tubP-GAL80, neoFRT 19A/neoFRT 19A; UAS-mCD8::GFP/+; tubP-GAL4/+* (WT) or *hsFLP, tubP-GAL80, neoFRT 19A/mei-41[G0221b] neoFRT 19A; UAS-mCD8::GFP/+; tubP-GAL4/+* (*mei-41* null mutant) were dissected and marked with anti-Dl (red), anti-GFP (green) antibodies and DAPI (blue) at 7 days after induction. The original magnification is 400×. (J) A graph showing the clone size of WT and *mei-41* mutant clone. The numbers of cells of each clone were counted in the posterior region of 9-10 midguts. ***p < 0.0001.

Surprisingly, most *esg*^+^ cells in the posterior midgut of *esg^ts^>GFP+ATMi+ATRi* and *esg^ts^>GFP+ATRi* flies disappeared after culture at 29 °C for 2 weeks (Figure 5A, yellow dotted region) and in the gut of *esg^ts^>GFP+ATMi* flies after culture at 29 °C for 3 weeks (data not shown). In the gut of *esg^ts^>GFP+ATRi*, *esg^ts^>GFP+ATMi*, and *esg^ts^>GFP+ATMi+ATRi* flies kept at 29 °C for 7 days, the Dl levels in *esg*^+^ cells *esg^ts^>GFP+ATMi+ATRi* and *esg^ts^>GFP+ATRi* were reduced markedly, and *esg*^+^ cells were larger and more spherical, compared to their counterparts in the gut of *esg^ts^>GFP* flies (Figure 5B). Under the same conditions, the shape of *esg*^+^ cells in the gut of *esg^ts^>GFP+ATMi* flies was similar to that of the control (Figure 5B). The total number of cells in the same area decreased in the gut of *esg^ts^>GFP+ATMi+ATRi* and *esg^ts^>GFP+ATRi* flies (Figure 5C), but the ratio of *esg*^+^ cells to total cells was unchanged compared to the control (Figure 5D). The ratio of Dl^+^ cells (a marker of ISCs) to *esg*^+^ cells in the gut of *esg^ts^>GFP+ATMi+ATRi* and *esg^ts^>GFP+ATRi* flies was reduced to 2.31% and 10.65%, respectively, whereas that in the control was 41% (Figure 5E). The ratio Dl^+^ cells to *esg*^+^ cells in the gut of *esg^ts^>GFP+ATMi* flies was 40.38% (Figure 5E). These results indicated that ATM and ATR were required for ISC maintenance, and the dependence on ATR was stronger than the dependence on ATM.

To determine the roles of ATM and ATR in ISC proliferation, we first analyzed the pattern and ratio of 5-ethynyl-2′-deoxyuridine (EdU)^+^ cells to *esg*^+^ cells in the gut with an ISC/EB-specific knockdown of ATM, ATR, or both. When ATM, ATR, or both were repressed in ISCs/EBs of *esg^ts^>GFP* flies after culture at 29 °C for 7 days, the number of EdU^+^ cells was significantly decreased compared to the control (Figure 5F). The ratio of EdU^+^ to *esg*^+^ cells in the gut of 7-day-old *esg^ts^>GFP+ATMi+ATRi*, *esg^ts^>GFP+ATRi*, and *esg^ts^>GFP+ATMi* flies kept at 29 °C was 6.9%, 7.1%, and 16.6%, respectively, whereas that in the control was 41.51% (Figure 5G). These results indicated that ATM and ATR were necessary for DNA replication in ISCs/EBs, and the role of ATR was more important than that of ATM.

Next, we examined the effects of an ISC/EB-specific knockdown of ATM, ATR, or both on the increase of ISC proliferation by PQ treatment. In gut specimens from PQ-treated flies, the number of proliferating cells increased in control *esg^ts^>GFP* flies, but the PQ-induced increase of ISC proliferation was strongly attenuated in gut specimens with an ISC/EB-specific knockdown of both ATM and ATR (Figure 5H). The PQ-induced increase of ISC proliferation was also strongly attenuated in the gut specimens with an ISC/EB-specific knockdown of ATR, while being weakly reduced if at all in the gut specimens with an ISC/EB-specific knockdown of ATM (Figure 5H). These results indicated that ATM and ATR in ISC/EB were required for the PQ-induced increase in ISC proliferation, and the role of ATR was greater than that of ATM. Furthermore, to confirm the role of ATR in maintenance and proliferation of midgut ISCs, we generated clones homozygous for the null allele *mei-41* encoding ATR using the mosaic analysis with a repressible cell marker (MARCM) method. Clones generated by MARCM method were allowed to grow for 7 days and stained for the ISC marker Delta (Figure 5I and Figure Supplemental S4). The number of cells per one clone in the posterior midgut was counted. The size of *mei-41* null mutant clones was markedly reduced compare to that of control (Figure 5J). These results indicated that ATR was required for maintenance and proliferation of midgut ISCs.

Taken together, these results indicated that ATM and ATR in ISCs/EBs were required for ISC maintenance and proliferation, and the role of ATR was bigger than that of ATM.

## DISCUSSION

This study shows that ATM and ATR are required for ISCs homeostasis, where ATR seems to play a more important role than does ATM in ISCs and EBs.

We first documented ATM/ATR activation by monitoring the pS/TQ immunostaining signals, as a dependable indicator of ATM/ATR activation in the *Drosophila* midgut. Our data on γ-irradiation- and PQ-induced pS/TQ signals and γH2AvD (*Drosophila* homolog of γH2AX) in gut specimens with an ISC/EB-specific knockdown of ATM, ATR, or both confirmed that pS/TQ signals in ISCs/EBs of the adult *Drosophila* midgut depend more on ATR than on ATM. Cimprich & Cortez [8] showed that ATM activation is rapid regard-less of the cell cycle compared to the slow ATR response predominantly in S- and G2-phase cells. Therefore, the ATR dependency of pS/TQ signals in ISCs and EBs of the *Drosophila* midgut is most likely associated with ISC proliferation for intestinal regeneration [17].

The present study also shows that the pS/TQ signals in *Drosophila* ISCs/EBs increase with age and under intrinsic (*Cat^n1^*) and extrinsic (PQ) oxidative stresses [25-26]. Therefore, our data showing the age- and oxidative stress-related increase in pS/TQ signals as well as the ATR dependency of pS/TQ signals confirm the possibility of differential contributions of ATM and ATR to ISC homeostasis during adulthood. Our data show that an ISC/EB-specific knockdown of ATR, ATM, or both affects ISC maintenance and proliferation. All of the phenotypic changes elicited by the ATR knockdown are much more substantial compared to the ATM knockdown. Our data indicate that ATM and ATR are required for ISC maintenance and proliferation, for which the role of ATR is more crucial than that of ATM in high-turnover tissues such as the intestine. On the other hand, we cannot rule out the possibility of different levels of the knockdown of ATM and ATR in the gut under a Gal4 driver.

The evidence presented here is clear that the DDR-related factors ATM and ATR in *Drosophila* ISC play a prosurvival role. Aged *Drosophila* ISCs show increased proliferative activity, in spite of increased γH2AvD formation [24, 27]. The age-dependent increase in ISC proliferation may be associated with DNA repair capacity as well as the outcome of a DNA damage response. DDR activates checkpoints and signals for DNA repair and then either allows for re-entry into the cell cycle, into senescence, or cell death, depending on the outcome of DNA repair [28-29]. It is known that the presence or absence of functional p53 (leading to cell death by apoptosis) shapes the execution of DDR [30]. Tumor cells, which often lack wild-type p53, are known to depend solely on the G2 arrest and to undergo mitosis with DNA breaks; this process is known as adaptation [30]. Adaptation in the face of a high mutation risk but with a slight chance of survival has been observed in yeast and mammalian cells [28-29]. On the basis of these observations, *Drosophila* ISCs may be a useful model for studies of the molecular mechanisms underlying the prosurvival role of DDR: adaptation.

ATM and ATR share many biochemical and functional similarities in the DDR, but differences are also known [28]. ATM is primarily activated by DNA double-strand breaks [8], participates in the G1/S phase checkpoint and activates the p53 response to DNA damage [10]. ATR is activated by DNA replication stress [8] and serves as a key effector of the G2/M checkpoint [11]. Several recent reports demonstrated that ATM and ATR are involved in the prosurvival function of DDR [29, 31-32]. ATM is a positive regulator of the AKT pathway, a key mechanism of survival [33]. ATR controls cellular adaptation in hypoxic tumors [31]. In accordance with ATR's involvement in prosurvival pathway, ATR inhibition has been suggested as a new approach to increasing the sensitivity of tumor cells to radiochemotherapy [32]. Therefore, a better understanding of the network of ATM/ATR-mediated prosurvival pathways in *Drosophila* ISCs shed insights into not only cancer therapy, but also how stem cell homeostasis is regulated in an aging gut, as shown in the present study.

ISC proliferation in the intestine is linked to the lifespan of the whole organism [14]. We found that an ISC/EB-specific knockdown of both ATM and ATR not only affects ISC proliferation but also reduces the lifespan of the flies (Figure Supplemental S5). In flies with mutations in *tefu* (ortholog of mammalian ATM) or *mei-41* (ortholog of mammalian ATR), the lifespan is reportedly shortened compared to the wild-type [34-35]. In humans, patients with ataxia-telangiectasia generally die by the second or third decade of life [36]. Therefore, our data in this regard suggest that DDR in ISCs is linked to organismal aging.

## METHODS

### Fly stock

Fly stocks were maintained at 25 °C on standard feed in the approximate 12 h/12 h light/dark cycle. The feed consisted of 79.2% of water, 1% of agar, 7% of cornmeal, 2% of yeast, 10% of sucrose, 0.3% of bokinin and 0.5% of propionic acid. To avoid larval overpopulation in all vials, 50–60 adult flies per vial were transferred to new food vials every 2–3 days for a period of 50–60 days or longer. We used the following transgenic RNAi strains of flies: *UAS-ATM-RNAi^22502^* (#22502, VDRC, Vienna, Austria), *UAS-ATM-RNAi^108074^* (#108074, VDRC), *UAS-ATR-RNAi^11251^* (#11251, VDRC), and *UAS-ATR-RNAi^103624^* (#103624, VDRC). The *esg-GAL4,UAS-GFP/CyO* flies were provided by the Drosophila Genetic Resource Center (DGRC, Kyoto, Japan). Temperature-inducible ISC/EB-specific *esg-Gal80^ts^* flies [17] were obtained from B. Ohlstein*.* Catalase mutant (*Cat^n1^*) flies were provided by the Bloomington Drosophila Stock Center (BDSC). *Oregon-R* flies served as the wild type. The *esg^ts^>GFP* flies were the result of a cross of the *Oregon-R* males and *esg-GAL4,UAS-GFP,tub-Gal80^ts^/CyO* (*esg^ts^*) females*.* The *esg^ts^>GFP+ATMi+ATRi* flies were obtained by crossing the *UAS-ATRi/UAS-ATRi*;*UAS-ATMi/UAS-ATMi* males and *esg^ts^ females*. For MARCM, we used P{ry[+t7.2]=hsFLP}1, P{w[+mC]=tubP-GAL80}LL1 w[*] P{ry[+t7.2]=neoFRT}19A; P{w[+mC]=UAS-mCD8::GFP.L}LL5 (#5134, BDSC), y[1] w[*]; P{w[+mC]=tubP-GAL4}LL7/TM3, Sb[1] Ser[1] (#5138, BDSC), *P{ry[+t7.2]=neoFRT}19A; ry*[506] (#1709, BDSC), and y[1] w[*] P{w[+mC]=lacW}G0221a P{lacW}mei-41[G0221b], l(1)G0221[G0221] P{neoFRT}19A/FM7c; P{ey-FLP.N}5 (#111381, DGRC). The results described in this study were obtained using female flies.

### MARCM

Wild-type (WT) MARCM clones were induced by heat-shock for 45 min in 37 °C water bath in female flies of the genotype *hsFLP, tubP-GAL80, neoFRT 19A/neoFRT 19A; UAS-mCD8::GFP/+; tubP-GAL4/+*. The *mei-41* MARCM clones were induced as described above in the genotypes *hsFLP, tubP-GAL80, neoFRT 19A/mei-41[G0221b] neoFRT 19A; UAS-mCD8::GFP/+; tubP-GAL4/+* [37]. To assess cell lineage, markers were analyzed at 7 day after induction. Clonal composition was counted from posterior region in 9-10 midguts of each genotype.

### Immunochemical analysis

An intact adult gut was dissected and fixed at room temperature. For staining with an anti-green fluorescent protein (GFP) antibody, the gut was fixed for 1 h in 4% formaldehyde (Sigma-Aldrich, St. Louis, MO, USA). For staining with anti-γH2AvD and anti-Dl antibodies, the whole-gut specimens were fixed for 30 min in 4% paraformaldehyde (Electron Microscopy Science, USA), dehydrated for 5 min in 50%, 75%, 87.5%, and 100% methanol, and rehydrated for 5 min in 50%, 25%, and 12.5% methanol in PBST (0.1% Triton X-100 in phosphate-buffered saline) for postfixing. Then, the specimens were washed with PBST and incubated overnight with a primary antibody at 4 °C. After washing with PBST, we incubated the specimens for 1 h with secondary antibodies at 25 °C, washed the specimens again in PBST, mounted them on Vectashield (Vector Laboratories, Burlingame, CA, USA), and analyzed them under a Zeiss Axioskop 2Plus microscope (Carl Zeiss Inc., Göttingen, Germany). For quantitative analysis of pS/TQ and γH2AvD, the images were processed in Photoshop (Adobe Systems Incorporated, San Jose, CA, USA). Wild-type cells and *esg^+^*, Delta protein*^+^* (DI^+^), pS/TQ*^+^*, and EdU*^+^* cells were counted in a 0.06 × 0.04-cm area of the posterior midgut. PH3^+^ cells were counted in the entire midgut.

### Antisera

The following primary antibodies (diluted in PBST) were used in our experiments: mouse anti-Dl, mouse anti-Arm (Developmental Studies Hybridoma Bank, Iowa City, IA, USA), 1:200; mouse anti-GFP and rabbit anti-GFP (Molecular Probes, Eugene, OR, USA), 1:1000; rabbit anti-γH2AvD (Rockland, Gilbertsville, PA, USA), 1:2000; rabbit anti-pS/TQ (Cell Signaling Technologies, Danvers, MA, USA), 1:1000; rabbit anti-phospho-histone H3 (PH3, Millipore, Billerica, MA, USA), 1:1000; mouse anti-γ-tubulin (Sigma-Aldrich, St. Louis, MO, USA), at 1:1000 dilution. The following secondary antibodies (diluted in PBST) were used: a goat anti-rabbit fluorescein isothiocyanate (FITC) conjugate (Cappel, Solon, OH, USA), 1:400; a goat anti-rabbit Cy3 conjugate (Jackson ImmunoResearch, West Grove, PA, USA), 1:400; a goat anti-mouse FITC conjugate (Jackson ImmunoResearch), 1:400; and a goat anti-mouse Cy3 conjugate (Jackson ImmunoResearch), 1:400. We also used 4′,6-diamidino-2-phenylindole (DAPI, Molecular Probes) for staining, at a 1:1000 dilution.

### γ-Irradiation

Adult flies were irradiated using a γ-ray machine [^137^Cs, 21.275 tBq (575 Ci)] at the dose-rate of 2.55 Gy/min. After irradiation at the 5 Gy dose, the irradiated flies and unirradiated control fly vials were maintained at 25 °C [24].

### PQ treatment

Flies were starved for 2 h prior to placement in empty in 10 mM PQ with 5% sucrose. After incubation at 25 °C for 18-20 h, whole-gut specimens were excised and analyzed by immunostaining.

### 5-Ethynyl-2′-deoxyuridine (EdU) incorporation

For Edu labeling, flies were starved for 2 h before being placed in vials containing 2.5 × 3.75 cm filter paper soaked in 100 μM EdU (Invitrogen, Grand Island, NY, USA) with 5% sucrose for 24 h. The gut was excised and fixed with 4% formaldehyde for 20 min. EdU incorporation was quantified using an EdU Alexa Fluor 488 Heat Shock Assay (Click-iT, Invitrogen) according to the manufacturer's instructions. After EdU incorporation, the whole-gut specimens were subjected to anti-Dl antibody staining as described previously [17, 38].

### Analysis of pS/TQ signals

Fluorescent images were captured using the same exposure time in each experiment by means of a Zeiss AxioSkop 2 Plus microscope using the AxioVision Rel 4.8 software (Carl Zeiss Inc.). Individual pS/TQ signals could not be distinguished accurately in the gut of young flies. Therefore, Adobe Photoshop CS5.1, extended version (Adobe System Incorporated), was used to measure the fluorescence level of pS/TQ. Individual merged fluorescent images were split in three channels, and the specific channel for pS/TQ was used to analyze the fluorescence levels. Fluorescence levels were measured within the nucleus using the DAPI channel based on the boundaries defined using the Photoshop CS5 quick-selection tool. Mean fluorescence was analyzed after subtraction of the mean fluorescence of the background region (from three spots excluding the nuclear portion in the posterior midgut). The mean fluorescence of the background region was not significantly different from that for secondary-antibody-only controls (data not shown). The mean fluorescence of the background was used to determine the fluorescence threshold. The data were analyzed in Sigma Plot 10.0 (Systat Software Inc., San Jose, CA, USA). Fluorescence measurements are presented in arbitrary units. We excluded the pS/TQ signals of mitotic cells; these signals were strong in the gut of not only young but also aged flies (Figure Supplemental S1B).

### Quantitative analysis of the cell number

For quantitative analysis of PH3-positive cells, we counted the number of such cells in the whole gut. After that, the total numbers of cells and GFP-, Dl-, or EdU-positive cells were evaluated in a visual field of the posterior midgut.

### Statistical analysis

Quantified data are expressed as the mean ± SE. Significance testing was conducted using Student's *t* test.

## SUPPLEMENTARY METHODS, FIGURES



## References

[1] Florian MC, Geiger H (2010). Concise review: polarity in stem cells, disease, and aging. Stem Cells.

[2] Nagaria P, Robert C, Rassool FV (2013). DNA double-strand break response in stem cells: mechanisms to maintain genomic integrity. Biochim Biophys Acta.

[3] Kim GD, Choi YH, Dimtchev A, Jeong SJ, Dritschilo A, Jung M (1999). Sensing of ionizing radiation-induced DNA damage by ATM through interaction with histone deacetylase. J Biol Chem.

[4] Schwartz MF, Duong JK, Sun Z, Morrow JS, Pradhan D, Stern DF (2002). Rad9 phosphorylation sites couple Rad53 to the Saccharomyces cerevisiae DNA damage checkpoint. Mol Cell.

[5] Dar I, Biton S, Shiloh Y, Barzilai A (2006). Analysis of the ataxia telangiectasia mutated-mediated DNA damage response in murine cerebellar neurons. J Neurosci.

[6] Vermezovic J, Stergiou L, Hengartner MO F (2012). Differential regulation of DNA damage response activation between somatic and germline cells in Caenorhabditis elegans. Cell Death Differ.

[7] Zhou J, Lim CU, Li JJ, Cai L, Zhang Y (2006). The role of NBS1 in the modulation of PIKK family proteins ATM and ATR in the cellular response to DNA damage. Cancer Lett.

[8] Cimprich KA, Cortez D (2008). ATR: an essential regulator of genome integrity. Nat Rev Mol Cell Biol.

[9] Kastan MB, Bartek J (2004). Cell-cycle checkpoints and cancer. Nature.

[10] Chen L, Gilkes DM, Pan Y, Lane WS, Chen J (2005). ATM and Chk2-dependent phosphorylation of MDMX contribute to p53 activation after DNA damage. EMBO J.

[11] Smith J, Tho LM, Xu N, Gillespie DA (2010). The ATM-Chk2 and ATR-Chk1 pathways in DNA damage signaling and cancer. Adv Cancer Res.

[12] Brown EJ, Baltimore D (2000). ATR disruption leads to chromosomal fragmentation and early embryonic lethality. Genes Dev.

[13] Dart DA, Adams KE, Akerman I, Lakin ND (2004). Recruitment of the cell cycle checkpoint kinase ATR to chromatin during S-phase. J Biol Chem.

[14] Rera M, Azizi MJ, Walker DW (2013). Organ-specific mediation of lifespan extension: more than a gut feeling?. Ageing Res Rev.

[15] Micchelli CA, Perrimon N (2006). Evidence that stem cells reside in the adult Drosophila midgut epithelium. Nature.

[16] Ohlstein B, Spradling A (2006). The adult Drosophila posterior midgut is maintained by pluripotent stem cells. Science.

[17] Ohlstein B, Spradling A (2007). Multipotent Drosophila intestinal stem cells specify daughter cell fates by differential notch signaling. Science.

[18] Lucchetta EM, Ohlstein B (2012). The Drosophila midgut: a model for stem cell driven tissue regeneration. Wiley Interdiscip Rev Dev Biol.

[19] Choi NH, Kim JG, Yang DJ, Kim YS, Yoo MA (2008). Age-related changes in Drosophila midgut are associated with PVF2, a PDGF/VEGF-like growth factor. Aging Cell.

[20] Biteau B, Hochmuth CE, Jasper H (2008). JNK activity in somatic stem cells causes loss of tissue homeostasis in the aging Drosophila gut. Cell Stem Cell.

[21] Park JS, Kim YS, Yoo MA (2009). The role of p38b MAPK in age-related modulation of intestinal stem cell proliferation and differentiation in Drosophila. Aging.

[22] Ayyaz A, Jasper H (2013). Intestinal inflammation and stem cell homeostasis in aging Drosophila melanogaster. Front Cell Infect Microbiol.

[23] Park JS, Pyo JH, Na HJ, Jeon HJ, Kim YS, Arking R, Yoo MA (2014). Increased centrosome amplification in aged stem cells of the Drosophila midgut. Biochem Biophys Res Commun.

[24] Park JS, Lee SH, Na HJ, Pyo JH, Kim YS, Yoo MA (2012). Age- and oxidative stress-induced DNA damage in Drosophila intestinal stem cells as marked by Gamma-H2AX. Exp Gerontol.

[25] Griswold CM, Matthews AL, Bewley KE, Mahaffey JW (1993). Molecular characterization and rescue of acatalasemic mutants of Drosophila melanogaster. Genetics.

[26] Bus JS, Gibson JE (1984). Paraquat: model for oxidant-initiated toxicity. Environ Health Perspect.

[27] Na HJ, Park JS, Pyo JH, Lee SH, Jeon HJ, Kim YS, Yoo MA (2013). Mechanism of metformin: Inhibition of DNA damage and proliferative activity in Drosophila midgut stem cell. Mech Aging Dev.

[28] Bartek J, Lukas J (2007). DNA damage checkpoints: from initiation to recovery or adaptation. Curr Opin Cell Biol.

[29] Swift LH, Golsteyn RM (2014). Genotoxic anti-cancer agents and their relationship to DNA damage, mitosis, and checkpoint adaptation in proliferating cancer cells. Int J Mol Sci.

[30] Caputo F, Vegliante R, Ghibelli L (2012). Redox modulation of the DNA damage response. Biochem Pharmacol.

[31] Fallone F, Britton S, Nieto L, Salles B, Muller C (2013). ATR controls cellular adaptation to hypoxia through positive regulation of hypoxia-inducible factor 1 expression. Oncogene.

[32] Fokas E, Prevo R, Pollard JR, Reaper PM, Charlton PA, Cornelissen B, Vallis KA, Hammond EM, Olcina MM, Gillies McKenna W, Muschel RJ, Brunner TB (2012). Targeting ATR in vivo using the novel inhibitor VE-822 results in selective sensitization of pancreatic tumors to radiation. Cell Death Dis.

[33] Hawkins AJ, Golding SE, Khalil A, Valerie K (2011). DNA double-strand break — induced pro-survival signaling. Radiother Oncol.

[34] Zainullin VG, Moskalev AA (2000). Effect of chronic low-dose irradiation and etoposide on the life spain of Drosophila melanogaster strain mei-41. Genetika.

[35] Moskalev AA, Plyusnina EN, Shaposhnikov MV (2011). Radiation hormesis and radioadaptive response in Drosophila melanogaster flies with different genetic backgrounds: the role of cellular stress-resistance mechanisms. Biogerontology.

[36] Barlow C, Hirotsune S, Paylor R, Liyanage M, Eckhaus M, Collins F, Shiloh Y, Crawley JN, Ried T, Tagle D, Wynshaw-Boris A (1996). Atm-deficient mice: a paradigm of ataxia telangiectasia. Cell.

[37] Lee T, Luo L (1999). Mosaic analysis with a repressible neurotechnique cell marker for studies of gene function in neuronal morphogenesis. Neuron.

[38] Amcheslavsky A, Jiang J, Ip YT (2009). Tissue damage-induced intestinal stem cell division in Drosophila. Cell Stem Cell.

