# Supplementary material for: Requirement of ATR for maintenance of intestinal stem cells in aging Drosophila
Source: Aging (Albany NY). 2015 May 12;7(5):307–18. doi: 10.18632/aging.100743 (PMC4468312; doi:10.18632/aging.100743)

Supplemental Figure. S1

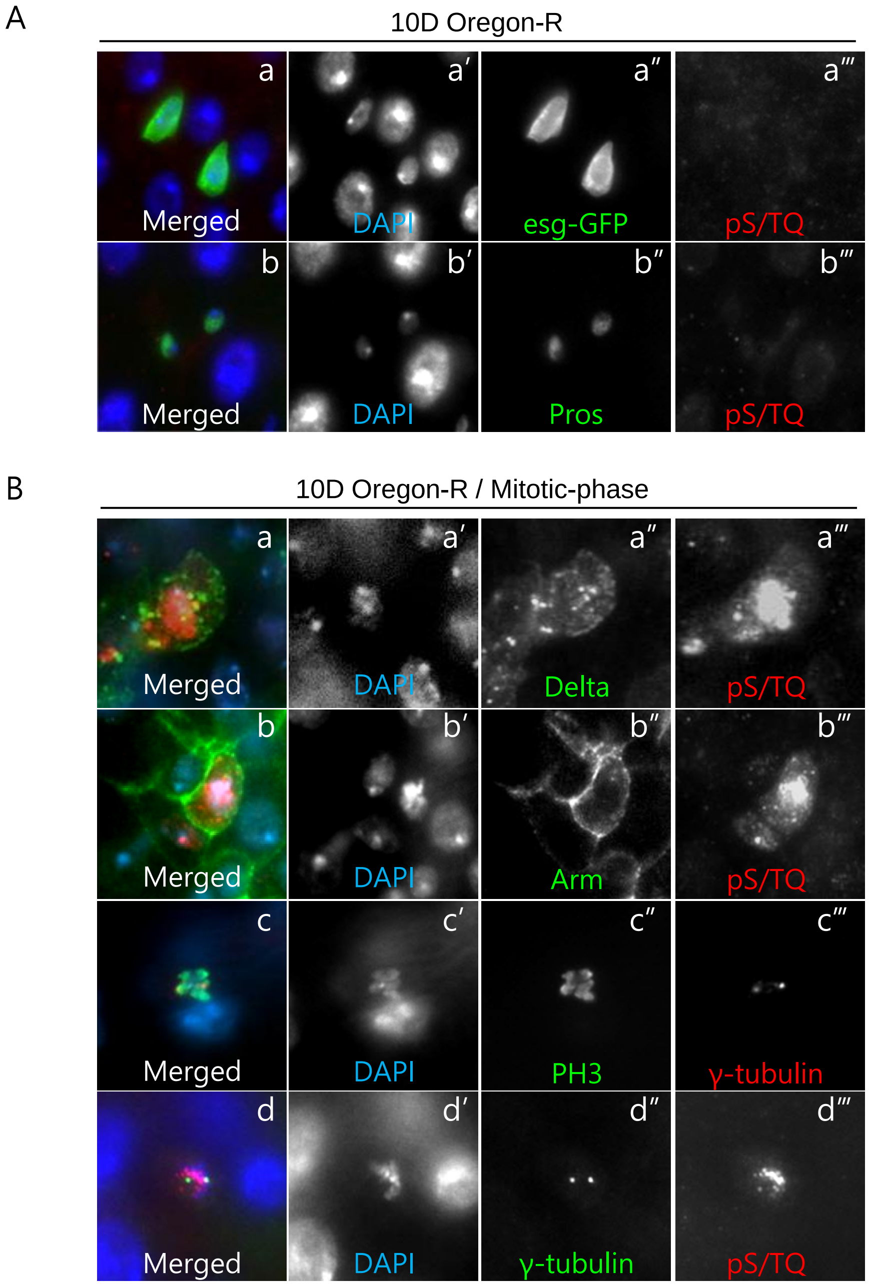

Supplemental Figure. S2

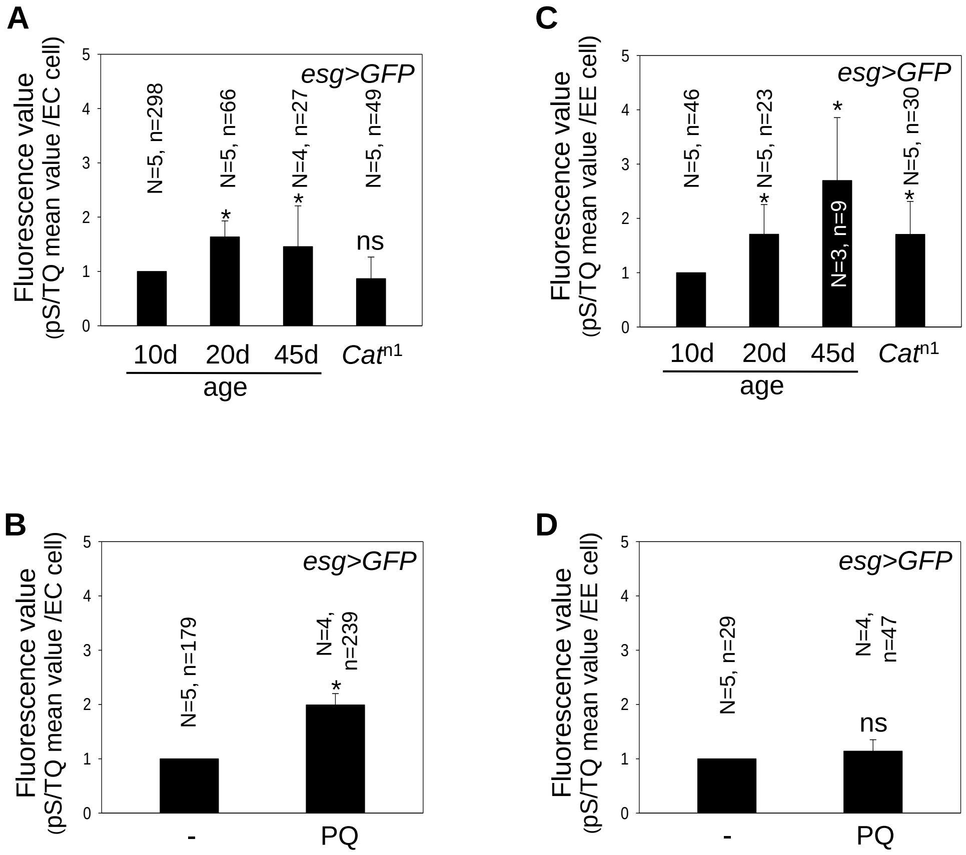

Supplemental Figure. S3

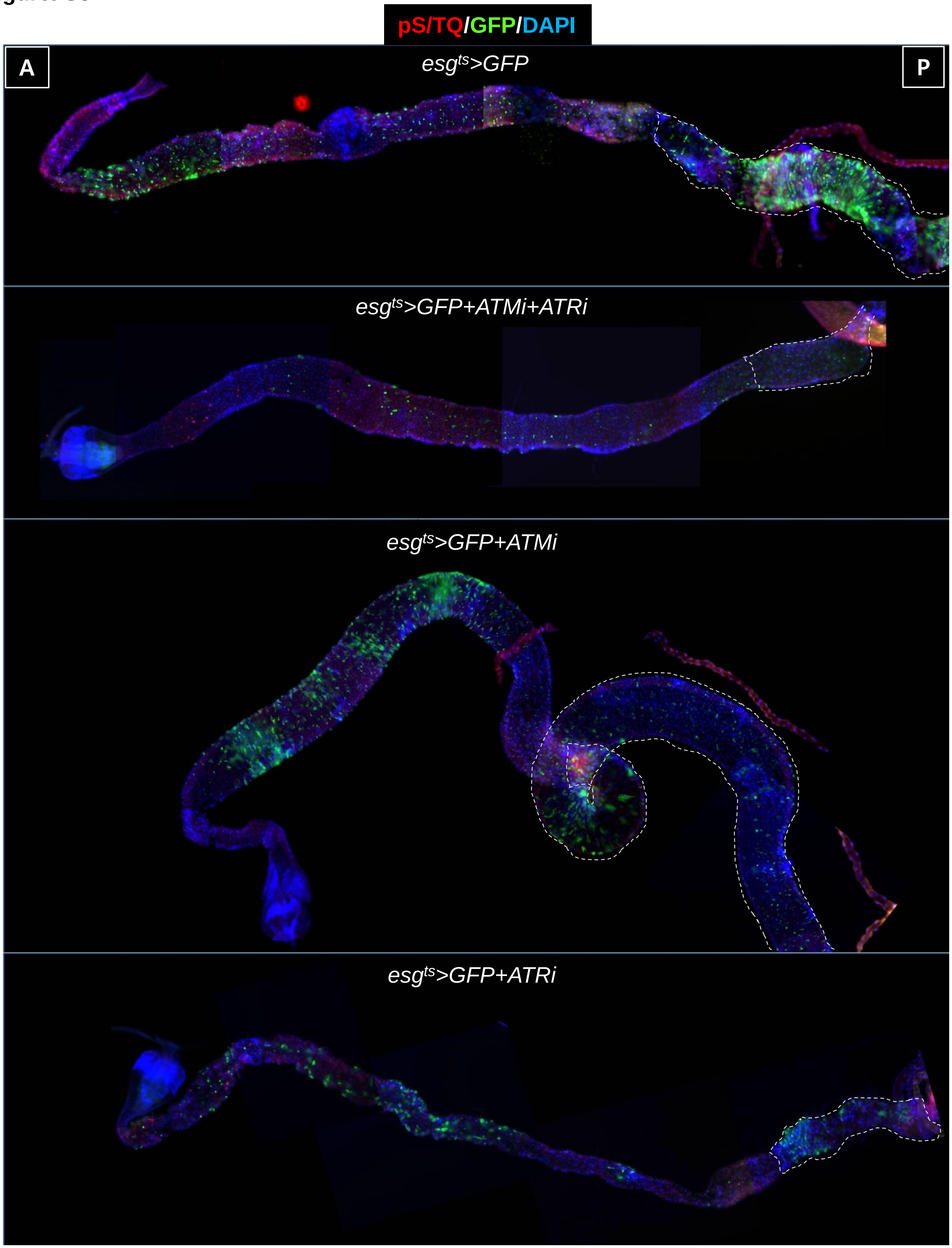

Supplemental Figure. S4

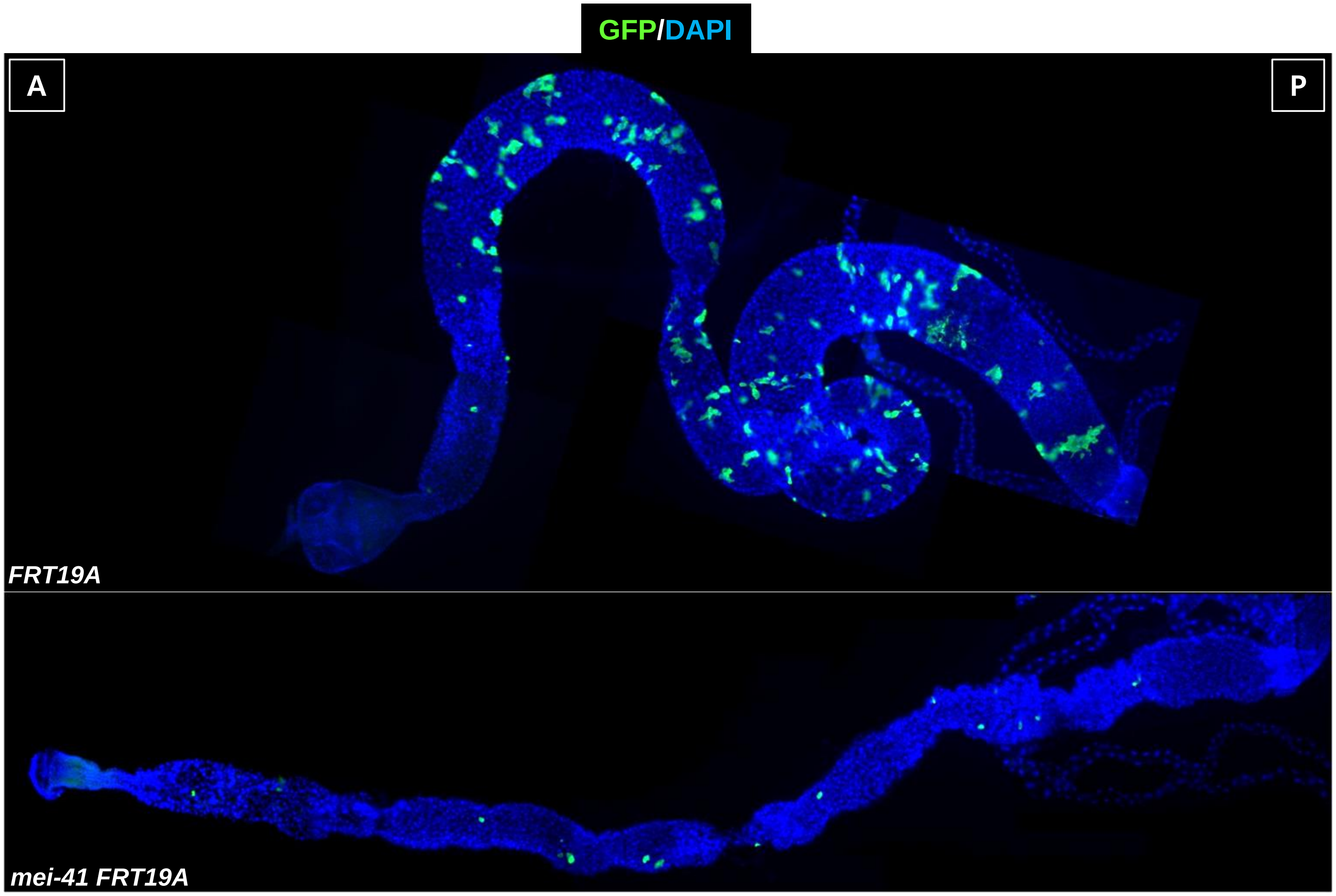

Supplemental Figure. S5

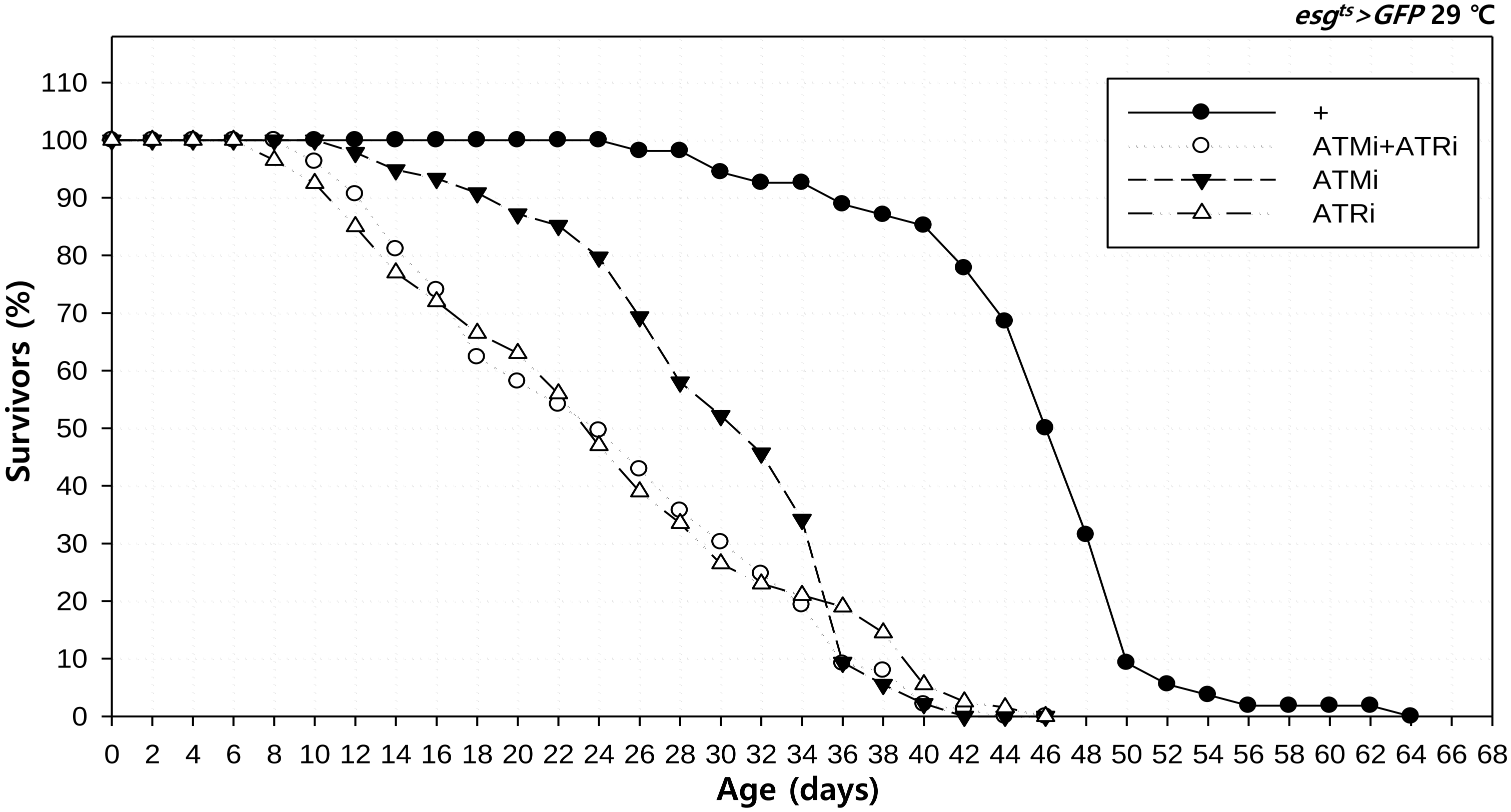

Supplement: Supplementary file 1 [file aging-07-0307-s001.pdf]
